# Hepatoprotective effect of Matrine salvianolic acid B salt on Carbon Tetrachloride-Induced Hepatic Fibrosis

**DOI:** 10.1186/1476-9255-9-16

**Published:** 2012-05-04

**Authors:** Hong-Ying Gao, Guo-Yu Li, Meng-Meng Lou, Xiao-Yu Li, Xiu-Yan Wei, Jin-Hui Wang

**Affiliations:** 1 School of Pharmacy, Shihezi University, Shihezi 832002, P. R. China; 2 School of Traditional Chinese Materia Medica 49#, Shenyang Pharmaceutical University, Wenhua Road 103, Shenyang 110016, P. R. China; 3 Key Laboratory of Phytomedicine Resources & Modernization of TCM, Shihezi 832002, P. R. China

**Keywords:** Carbon tetrachloride, Hepatic fibrosis, Matrine salvianolic acid B salt

## Abstract

The aim of this study was to investigate the hepatoprotective effect of Matrine salvianolic acid B salt on carbon tetrachloride (CCl_4_)-induced hepatic fibrosis in rats. Salvianolic acid B and Matrine has long been used to treat liver fibrosis. Matrine salvianolic acid B salt is a new compound containing Salvianolic acid B and Matrine. Hepatic fibrosis induced by CCl_4_ was studied in animal models using Wistar rats. Organ coefficient, serum aspartate aminotransferase (AST), alanine aminotransferase (ALT), hexadecenoic acid (HA), laminin (LN), hydroxyproline (Hyp), and glutathione (GSH), malondialdehyde (MDA), superoxide dismutase (SOD) in liver tissues were measured, respectively. Histopathological changes in the livers were studied by hematoxylin-eosin (H&E) staining and Masson Trichrome (MT) examination. The expression of transforming growth factor-β_1_ (TGF-β_1_) and α-smooth muscle actin (α-SMA) was observed by immunohistochemical analysis. A significant reduction in serum levels of AST, ALT, HA, LN and Hyp was observed in the Matrine salvianolic acid B salt treated groups, suggesting that the salt had hepatoprotective effects. The depletion of GSH and SOD, as well as MDA accumulation in liver tissues was suppressed by Matrine salvianolic acid B salt too. The expression of TGF-β_1_ and α-SMA measured by immunohistology was significantly reduced by Matrine salvianolic acid B salt in a dose-dependent manner. Matrine salvianolic acid B salt treatment attenuated the necro-inflammation and fibrogenesis induced by CCl_4_ injection, and thus it is promising as a therapeutic anti-fibrotic agent against hepatic fibrosis.

## Introduction

Hepatic fibrosis is a dynamic process characterized by excessive deposition of extracellular matrix (ECM) components, and can ultimately cause liver cirrhosis. Activation of hepatic stellate cells (HSCs) is the key step during the progress of hepatic fibrosis [1]. Upon activation by liver injuries, HSCs transform into Myofibroblastic cells which are proliferative and fibrogenic, with enhanced production of ECM components including α-smooth muscle actin (α-SMA), hexadecenoic acid (HA) and laminin (LN) [2]. Generally, CCl_4_ is metabolized by microsomal monooxygenase system (cytochrome P_450_ 2E1) to its active metabolite, this process results in the fragmentation of the lipid peroxide radicals, lipid hydroperoxides and other products, each acting like an active oxidizing agent [3-6]. Furthermore, these processes are immediately followed by the infiltration of inflammatory cells and release of various cytokines and growth factors [6]. Thus, lipid peroxidation caused by free radicals of CCl_4_ metabolism plays a vital role on the CCl_4_-induced liver injury [6,7]. In addition to lipid peroxidation, transforming growth factor β_1_ (TGF-β_1_) is also an important activator of HSCs in the course of hepatic fibrogenesis [3].

### *Sophora flavescens* ait

Has a wide range of pharmacological and toxicological activities [8]. This herb has been used traditionally in China against several pathophysiological states, such as cancer, viral hepatitis, cardiac arrhythmia, and asthma [9]. Matrine is one of the most important alkaloids extracted from this herb and it exerts a variety of pharmacological effects, such as anti-inflammatory, immunoregulatory, antivirus, anti-fibrosis and anti-tumor [10,11]. In recent years, Matrine has been used in the treatment of chronic liver disease and has a significant effect on the inhibition of liver fibrosis [12].

### *Salvia miltiorrhiza* Bge

Is a well-known traditional Chinese herb which is broadly planted in China. It has been used clinically for the treatment of various diseases such as cardiovascular, cerebrovascular, hyperlipidemia, and acute ischemic stroke diseases [13] and has increasingly attracted the attention of research groups in recent years especially in biotechnological field. Salvianolic acid B (Sal-B) is one of the water-soluble components of this herb. Previous studies have shown that Salvianolic acid B is effective in improving liver function, alleviating ischemic damage, antioxidation, antihepatotoxicity and anticoagulation [14]. At present, the pharmacology activity of the salvianolic acid B magnesium salt has been widely studied [15].

In order to obtain the cheap and highly efficient anti-fibrosis drug, we adopted salvianolic acid B and Matrine as raw materials to prepare Matrine salvianolic acid B salt, which is first reported in this article. The structural of Matrine salvianolic acid B salt has been confirmed by the technique of TLC, UV, IR, NMR and TG-DSC. Matrine salvianolic acid B salt has a good stability and can overcome the shortcomings of salvianolic acid B and Matrine [16]. Previous studies have shown its protective effect against acute hepatotoxicity by reducing serum AST and ALT levels induced by the treatment of CCl_4_, Thioacetamide(TAA) and D-Galactosamine (D-GalN) respectively in mice. The present study aimed to investigate the protective effects of Matrine salvianolic acid B salt on the liver fibrosis induced by CCl_4._ ( Additional file 1).

## Materials and methods

### Drug material

Matrine (blanc needle crystal, >98%) was obtained from Shan xi yi xing biological engineering limited company (Shanxi, China). Salvianolic acid B (amber powder, >95%) and Matrine salvianolic acid B salt (amber powder) were prepared by ourself. The voucher specimen (No. 20070816066) is deposited in School of Pharmacy faculty, Shihezi University.

### Animals

Wistar Albino rats weighing between 180 and 250 g were used with free access to food and water. Rats were housed at room temperature with 12 h light/dark cycle. The use of these animals was approved by the institute ethnics committee of Shihezi University. The chronic liver injury was induced by CCl_4_ injection as previously described [5-7]. Rats were randomized into eight groups (n = 12 in each group). The normal control group was allowed free access to food and water; Liver injury was performed in the remaining seven groups by i.p. 1.0 ml/kg CCl_4_ and olive oil (2:3 v/v) mixture, twice in a week for 8 weeks. The rats of the remaining seven groups except the model group were intragastric (i.g.) administrated quantum satis (q.s.) dosages respectively (Matrine salvianolic acid B salt group: 25 mg/kg, 50 mg/kg, 100 mg/kg respectively; Matrine control group: 50 mg/kg; Salvianolic acid B control group: 150 mg/kg; Positive control group: 780 mg/kg). Orally administration of different drugs were performed at the day before CCl_4_ administration, and applied once per day for 8 weeks.

Rats were sacrificed by cervical dislocation on the day after the last intragastrical administration. Blood and liver tissue samples were harvested for further examinations. Serum was obtained by the centrifuge at 3,000 rpm for 10 min under 4°C, and stored at −20°C before use. Liver tissues were collected for the measurements of glutathione (GSH), malondialdehyde (MDA), superoxide dismutase (SOD), as well as the histopathological changes.

### Biochemical analysis

At 12 h after last intragastrical administration, rats were killed. The blood sample were collected and then centrifuged at 3,000 rpm for 10 min at 4°C. Serum Aspartate aminotransferase (AST) and Alanine aminotransferase (ALT) were determined using Olympus kits (Olympus Corp., Tokyo, Japan) in an Olympus AU 600 Autoanalyzer. The observation absorbance of the reaction was read at 505 nm and the enzyme activity was calculated as U/L.

Serum Hexadecenoic acid (HA), Laminin (LN) and Hydroxyproline (Hyp) levels were measured by the enzyme-linked immunosorbent assay method using the kit obtained from Sigma-Aldrich Chemicals Co., USA. The observation absorbance of the reaction was read at 450 nm.

### Measurement of malondialdehyde (MDA) formation in lipid peroxidation

Liver homogenate (10%, w/v) was prepared by homogenizing the liver tissue in 150 mM Tris–HCl buffered saline (pH 7.2) with a polytron homogenizer. The level of MDA in liver tissues was measured by following the protocol provided by the kit from Jiancheng Biological Engineering Institute (Nanjing, China).

### Determination of antioxidant and antioxidant enzyme activity

SOD activity was determined by the commercial kit from Jiancheng Biological Engineering Institute (Nanjing, China) following the protocol provided by manufacture. Data are expressed as SOD U/mg protein. The measurement of GSH was conducted by modified protocol provided by GSH kit from Jiancheng Biological Engineering Institute (Nanjing, China). The observation absorbance of the reaction was read at 420 nm and the enzyme activity was calculated as mg/g protein. The protein content was measured by the methods of Lowry et al. (1951) with bovine serum albumin as a standard.

### Histopathological evaluation

Four-micrometer-thick sections obtained from paraffin blocks were stained by hematoxylin-eosin (H&E) and Masson trichrome (MT), and then examined with an Olympus BX-50 microscope by × 40, ×100, ×200, and × 400 magnifications. The histopathological evaluation was performed by an expert pathologist blinded to the study groups. The sections, stained with H&E, were examined by light microscopy by × 200 magnification. In the sections stained with MT, the central vein was focused, and the images randomly taken from ten fields were evaluated using image analysis, a specially designed software program by × 200 magnification. The liver damage was graded as following: _, absent; +, few; ++, mild and +++, moderate. Hepatic fibrosis was graded according to the method of Ruwart et al. (1989) [17] as the following: _, absent, normal liver; +, few, increase of collagen without formation of septa; ++, mild, formation of incomplete septa from portal tract to central vein (septa that do not interconnect with each other); and +++, moderate, complete but thin septa interconnecting with each other (incomplete cirrhosis). The final numerical score was calculated by dividing the sum of the number per grade of affected rat by the total number of examined rat.

### Immunohistological analysis

The activation of HSCs was identified by immunohistochemical analysis, staining by monoclonal α-SMA and TGF-β_1_ antibody in deparaffinized tissue sections. Paraffin section deparaffinage, use distilled water and 0.1 mol•L^−1^ PBS flush 5 min respectively; Then use 3% H_2_O_2_ incubation 10 min in room temperature, in order to deactivation endogenous peroxydase; Use 0.1 mol•L^−1^ PBS flush 5 min, three times, normo-goat serum 37°C blockage 30 min; Dropwise 1:50 diluted primary antibody 4°C to stay overnight, 0.1 mol•L^–1^ PBS flush 5 min, three times; Then dropwise 1:100 diluted biotinylation goat anti-rabbit secondary antibody, 37°C incubation 20 min; 0.1 mol•L^−1^ PBS flush 5 min, three times, dropwise SP, 37°C incubation 20 min; Use 0.1 mol•L^−1^ PBS flush 5 min, three times, DAB colouration; Distilled water flush, termination colouration; Hematoxylin counterstain; Gradient alcoholic dehydration, dimethyl benzene transparent and neutro-resin mounting. Observed under the microscope, the positive part shows Buffy.

### Statistical analysis

All Data were expressed as Means ± Standard Deviations, and analyzed with one-way analysis of variance (ANOVA). Statistical significance was analyzed by SPSS software using LSD’s test.

## Results

### The effect of Matrine salvianolic acid B salt on body weight decreasing induced by CCl_4_ treatment

**Figure 1:**
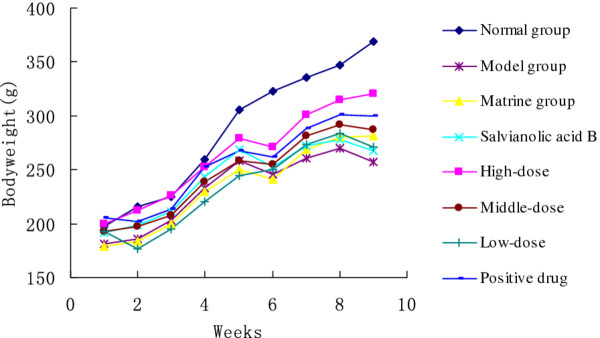
**The body weight of animals in the control, CCl**_**4**_**- and drug-treated groups.**

There was no significant difference among the mean initial body weights of all groups (*p* < 0.05, Figure 1). However, body weights of CCl_4_-and drug-treatment groups were lower than that of the control group by the end of the study (*p* < 0.05 for three doses of Matrine salvianolic acid B salt group, Figure 1). And there was no significant dose-respond relationship on the decreasing of body weight among drug-treatment groups (*p* < 0.05, Figure 1).

### The effect of Matrine salvianolic acid B salt on organ coefficient increasing induced by CCl_4_ treatment

Organ coefficients including liver, spleen and kidney coefficients were evaluated in rats. Similar to previous studies [18], liver and spleen coefficients were significantly increased in rats with CCl_4_-treatment (*p* < 0.01, Table 1); In contrast, there is no difference in kidney coefficient among all treated groups. As demonstrated in Table 1, the increase of liver coefficient caused by CCl_4_ treatment in rats was reduced by Salvianolic acid B (150 mg/kg), Matrine salvianolic acid B salt (25, 50, and 100 mg/kg), and Fu fang bie jia ruan gan pian treatment (780 mg/kg) individually (*p* < 0.05, Table 1), expect for Matrine treatment. Then the protective effect against the increase of spleen coefficient was observed in rats with Matrine (50 mg/kg), Matrine salvianolic acid B salt (25, 50 and 100 mg/kg) and Fu fang bie jia ruan gan pian treatment (780 mg/kg) (*p* < 0.01, Table 1), expect for Salvianolic acid B treatment (150 mg/kg). Dose-effect correlation was observed in Matrine salvianolic acid B salt groups regarding to the increase of liver and spleen coefficient by CCl_4_-treatment.

**Table 1:** Effect of Matrine salvianolic acid B salt on organ coefficient in hepotic fibrosis rats

Treatment	Doses (mg/kg)	Liver coefficient %	Spleen coefficient %	Kidney coefficient %
Normal control	——	2.55 ± 0.23	0.19 ± 0.05	0.73 ± 0.04
CCl_4_	——	3.53 ± 0.37^##^	0.27 ± 0.04^##^	0.71 ± 0.10
Matrine+ CCl_4_	50	3.33 ± 0.19	0.20 ± 0.03^**^	0.75 ± 0.19
Salvianolic acid B+ CCl_4_	150	3.14 ± 0.55^*^	0.26 ± 0.02	0.76 ± 0.16
	25	3.18 ± 0.46^*^	0.21 ± 0.03^**^	0.73 ± 0.13
Matrine salvianolic acid B Salt+ CCl_4_	50	3.17 ± 0.26^*^	0.20 ± 0.04^**^	0.71 ± 0.17
	100	3.16 ± 0.49^*^	0.19 ± 0.05^**^	0.67 ± 0.12
Fu fang bie jia ruan gan pian+ CCl_4_	780	3.16 ± 0.35^*^	0.20 ± 0.04^**^	0.72 ± 0.08

Data is expressed as the mean±SD (n = 12).

^##^p < 0.01 vs. the normal control group; ^*^p < 0.05, ^**^p < 0.01 vs. the CCl_4_-treated group.

### The effect of Matrine salvianolic acid B salt on the increase of serum AST and ALT in rats

There were dramatic increases of serum ALT and AST after CCl_4_ treatment in rats, which were 4 and 6 times higher compared to that of control group (*p* < 0.01, Table 2). Consistent with previous studies [19], Fu fang bie jia ruan gan pian treatment (780 mg/kg) showed a significantly protective effect against the increase of serum ALT and AST after long-term CCl_4_ injection in rats (*p* < 0.01, Table 2). Similarly, Matrine (50 mg/kg) and Salvianolic acid B treatment (150 mg/kg) decreased the elevation of serum ALT and AST to the level relevant to that of Fu fang bie jia ruan gan pian group after long-term CCl_4_ injection in rats (*p* < 0.01, Table 2). Furthermore, the decrease of serum ALT and AST in rats treated with CCl_4_ was observed in Matrine salvianolic acid B salt groups (25, 50 and 100 mg/kg), and the doses of Matrine salvianolic acid B salt 100 mg/kg showed a significantly protective effect (*p* < 0.01, Table 2).

**Table 2:** Effect of Matrine salvianolic acid B salt on AST and ALT in rats

Treatment	Doses (mg/kg)	AST (U/L)	ALT (U/L)
Normal control	——	45.59 ± 3.68	32.12 ± 14.09
CCl_4_	——	211.20 ± 42.07^##^	221.24 ± 42.35^##^
Matrine+ CCl_4_	50	142.87 ± 36.99^**^	138.87 ± 32.67^**^
Salvianolic acid B+ CCl_4_	150	104.22 ± 20.67^**^	154.92 ± 60.64^**^
Matrine salvianolic acid B Salt+ CCl_4_	25	159.10 ± 46.19^**^	169.92 ± 51.42^**^
	50	119.80 ± 25.69^**^	144.33 ± 44.93^**^
	100	102.49 ± 25.71^**b^	96.09 ± 27.28^**ac^
Fu fang bie jia ruan gan pian+ CCl_4_	780	110.3 ± 26.62^**^	117.26 ± 40.70^**^

Data is expressed as the mean±SD (n = 12).

^##^p < 0.01 vs. the normal control group; ^**^p < 0.01 vs. the CCl_4_-treated group;

^a^p < 0.05, ^b^p < 0.01 vs. the Matrine group; ^c^p < 0.01 vs. the Salvianolic acid B group.

### The effect of Matrine salvianolic acid B salt on the increase of serum HA, LN and Hyp in rats

After CCl_4_ admimistration, the levels of serum HA, LN and Hyp were significantly increased, compared with that of control group (*p* < 0.01, Table 3). However, treatment with Fu fang bie jia ruan gan pian (780 mg/kg) significantly decreased the levels of serum LN and HyP induced by CCl_4_ (*p* < 0.05, *p* < 0.01, Table 3), While there is no significant difference between the levels of HA. Meanwhile, Matrine (50 mg/kg) and Salvianolic acid B treatment (150 mg/kg) markedly decreased the elevation of serum HA, LN and HyP after long-term CCl_4_ injection in rats (*p* < 0.05, *p* < 0.01, Table 3). In addition, except the levels of LN in the group of 25 mg/kg Salt + CCl_4_, the levels of serum HA, LN and Hyp were all lower in the Matrine salvianolic acid B salt three doses group compared to CCl_4_-treated group (*p* < 0.05, *p* < 0.01, Table 3). Moreover, it has showed a good dose-effect relationship in various doses of Matrine salvianolic acid B salt.

**Table 3:** Effect of Matrine salvianolic acid B salt on HA, LN and Hyp in rats

Treatment	Doses (mg/kg)	HA (ng/ml)	LN (ng/ml)	HyP (ng/ml)
Normal control	——	213.46 ± 21.62	85.39 ± 3.98	16.38 ± 1.30
CCl_4_	——	328.71 ± 18.42^##^	109.93 ± 18.16^##^	32.34 ± 6.41^##^
Matrine+ CCl_4_	50	292.49 ± 30.73^*^	94.79 ± 19.24^*^	27.14 ± 1.58^**^
Salvianolic acid B+ CCl_4_	150	290.66 ± 53.77^*^	88.36 ± 19.44^**^	22.25 ± 1.88^**^
	25	289.88 ± 27.00^*^	99.79 ± 23.42	19.71 ± 1.52^**bc^
Matrine salvianolic acid B Salt+ CCl_4_	50	276.77 ± 47.19^**^	85.61 ± 14.21^**^	19.14 ± 1.86^**bd^
	100	264.70 ± 49.62^**^	81.61 ± 16.22^**a^	18.65 ± 1.94^**bd^
Fu fang bie jia ruan gan pian+ CCl_4_	780	299.76 ± 33.99	97.37 ± 15.35^*^	18.86 ± 1.79^**^

Data is expressed as the mean±SD (n = 12).

^##^p < 0.01 vs. the normal control group; ^*^p < 0.05, ^**^p < 0.01 vs. the CCl_4_-treated group;

^a^p < 0.05, ^b^p < 0.01 vs. the Matrine group; ^c^p < 0.05, ^d^p < 0.01 vs. the Salvianolic acid B group.

### The effect of Matrine salvianolic acid B salt on liver tissue GSH, MDA and SOD levels in rats

GSH and SOD could scavenge the lipid peroxide radicals, lipid hydroperoxides and other products which is a toxic metabolite of the CCl_4_. Therefore, we measured the contents of GSH and SOD in liver tissue of rats. From Table 4, we can clearly see the vast difference between CCl_4_-treated group and the control group, the levels of GSH and SOD were largely decreased (*p* < 0.01, Table 4) in CCl_4_-treated group compared with that of control group. The levels of GSH in Fu fang bie jia ruan gan pian treatment had significantly increased (*p* < 0.01, Table 4), while there was no significant difference in the level of SOD compared to the group of CCl_4_. After Salvianolic acid B treatment, The levels of GSH and SOD were higher than CCl_4_ treatment, but no significant difference. However, treatment with Matrine salvianolic acid B salt (25, 50 and 100 mg/kg) significantly recovered the CCl_4_-induced GSH and SOD depletion (*p* < 0.05, *p* < 0.01, Table 4). In addition, CCl_4_ rats exhibited a significant increase in hepatic MDA (a marker of lipid peroxidation levels) (*p* < 0.01, Table 4), when compared with the control group. On the other hand, treatment with Matrine salvianolic acid B salt (25, 50 and 100 mg/kg) showed a significant decrease in MDA levels with a dose-dependent manner, to 2.58 ± 0.62, 2.11 ± 0.31 (*p* < 0.01, Table 4) and 1.79 ± 0.43 (*p* < 0.01, Table 4) respectively.

**Table 4:** Effect of Matrine salvianolic acid B salt on GSH, MDA and SOD in rats

Treatment	Doses (mg/kg)	GSH (mg/gprotein)	MDA (nmol/mgprotein)	SOD (U/mg protein)
Normal control	——	3.00 ± 0.14	1.03 ± 0.22	262.95 ± 30.43
CCl_4_	——	1.85 ± 0.28^##^	3.00 ± 0.45^##^	151.95 ± 20.25^##^
Matrine + CCl_4_	50	2.36 ± 0.53^**^	2.52 ± 0.87	151.74 ± 11.44
Salvianolic acid B + CCl_4_	150	2.16 ± 0.65	2.12 ± 0.43^**^	159.85 ± 22.28
	25	2.28 ± 0.59*	2.58 ± 0.62	169.94 ± 25.16
Matrine salvianolic acid B Salt + CCl_4_	50	2.48 ± 0.33^**^	2.11 ± 0.31^**^	184.65 ± 24.90^**ab^
	100	2.63 ± 0.29^**b^	1.79 ± 0.43^**a^	191.71 ± 48.32^**ac^
Fu fang bie jia ruan gan pian + CCl_4_	780	2.52 ± 0.32^**^	1.76 ± 0.76^**^	171.39 ± 28.90

Data is expressed as the mean±SD (n = 12).

^##^p < 0.01 vs. the normal control group; ^*^p < 0.05, ^**^p < 0.01 vs. the CCl_4_-treated group;

^a^p < 0.01 vs. the Matrine group; ^b^p < 0.05, ^c^p < 0.01 vs. the Salvianolic acid B group.

### The effect of Matrine salvianolic acid B salt on histopathological evaluation

**Figure 2:**
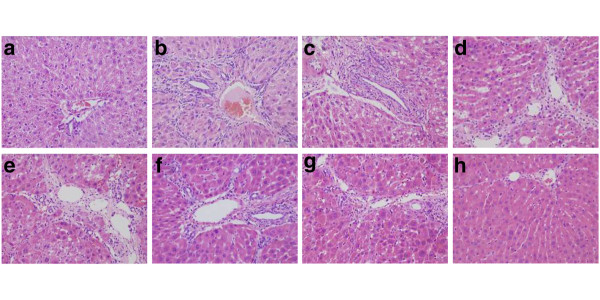
**The H&E (×200) of the liver sections in rats.** (**a**) Normal group; (**b**) CCl_4_-treated group; (**c**) Matrine + CCl_4_; (**d**) Salvianolic acid B + CCl_4_; (**e**), (**f**) and (**g**) are Matrine salvianolic acid B salt group treated with 25, 50 and 100 mg/kg respectively; (**h**) Positive-drug + CCl_4_.

**Figure 3:**
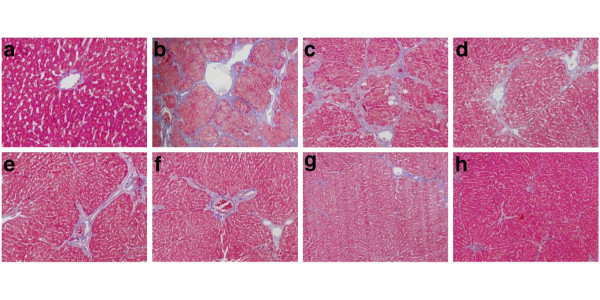
**The MT examinations (×200) of the liver sections in rats.** (**a**) Normal group; (**b**) CCl_4_-treated group; (**c**) Matrine + CCl_4_; (**d**) Salvianolic acid B + CCl_4_; (**e**), (**f**) and (**g**) are Matrine salvianolic acid B salt group treated with 25, 50 and 100 mg/kg respectively; (**h**) Positive-drug + CCl_4_.

In normal control group, liver sections showed normal hepatic cells with well preserved cytoplasm, prominent nucleolus and central vein (Figure 2a). The liver sections of CCl_4_ treated group showed a moderate degree of centrilobular necrosis, and mild degree of leukocytes infiltration, bile duct proliferation, mitosis and calcification (Figure 2b). The histological pattern of the livers treated by Matrine salvianolic acid B salt showed a few to milder degree of infiltration of leukocytes, absent to few degree of necrosis, and bile duct proliferation (Figure 2e, f and g). Similar trends were also observed in group of Fu fang bie jia ruan gan pian (Figure 2h). The histological observations also supported the results obtained from the serum enzyme assay. Further, histopathological changes of fibrosis occurred in CCl_4_-intoxicated and prevention by the treatment with Matrine salvianolic acid B salt are shown in Figure 3 The livers of rat treated with CCl_4_ showed extensive accumulation of connective tissue resulting in formation of continuous fibrotic septa, nodules of regeneration, and noticeable alterations in the central vein as compared to the normal control (Figure 3a and b). The group treated with Matrine salvianolic acid B salt and Fu fang bie jia ruan gan pian resulted in less pronounced destruction of the liver architecture without fibrosis (Figure 3e, f, g and h). According to microscopic examinations, severe hepatic fibrosis induced by CCl_4_ was remarkably reduced by the administration of Matrine salvianolic acid B salt, which was in good correlation with the results of the serum aminotransferase activities and hepatic antioxidant enzyme activities.

### The effect of Matrine salvianolic acid B salt on immunohistochemistry analisis

**Figure 4:**
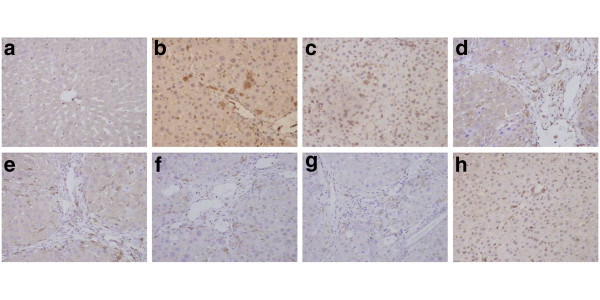
**The immunohistochemical examination of α-SMA positive cells (×400) of the liver sections in rats.** (**a**) Normal group; (**b**) CCl_4_ -treated group; (**c**) Matrine + CCl_4_; (**d**) Salvianolic acid B + CCl_4_; (**e**), (**f**) and (**g**) are Matrine salvianolic acid B salt group treated with 25, 50 and 100 mg/kg respectively; (**h**) Positive-drug + CCl_4_.

**Figure 5:**
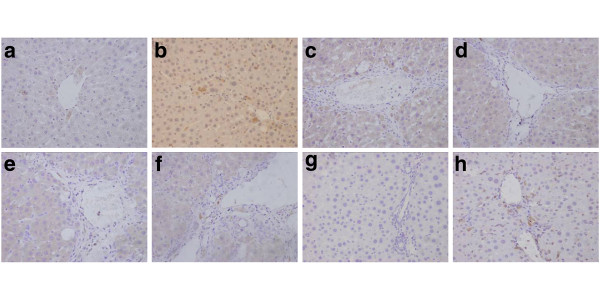
**The immunohistochemical examination of TGF-β**_**1**_**positive cells (×400) of the liver sections in rats.** (**a**) Normal group; (**b**) CCl_4_-treated group; (**c**) Matrine + CCl_4_; (**d**) Salvianolic acid B + CCl_4_; (**e**), (**f**) and (**g**) are Matrine salvianolic acid B salt group treated with 25, 50 and 100 mg/kg respectively; (**h**) Positive-drug + CCl_4_.

Histological examination of livers from CCl_4_ rats revealed the increase and expansion of fibrous septa, compared with normal control rats. α-SMA and TGF-β_1_ expression were more distinctly observed in the liver of CCl_4_ rats as compared with normal control rats (Figure 4a, b and Figure 5a, b). Treatment with Matrine salvianolic acid B salt decreased α-SMA and TGF-β_1_ staining (Figure 4e, f, g and Figure 5e, f, g). The fibrosis score in CCl_4_ rats was significantly increased as compared to that of control rats, and treatment of Matrine salvianolic acid B salt significantly ameliorated hepatic fibrosis in CCl_4_ rats. Similarly, protein components of α-SMA and TGF-β_1_ were significantly down-regulated in the group of Matrine, Salvianolic acid B and Fu fang bie jia ruan gan pian. Moreover, ascite was detected in three rats from the CCl_4_-treated group with an odds ratio, while it was not observed in the other groups (Figures 4 and 5).

## Discussion

CCI_4_-induced toxic liver injury which is a well characterized model for hepatic fibrosis has been extensively performed [5,6]. Hepatic fibrosis is a dynamic cascade beginning with hepatocyte necrosis followed by the activation of the inflammatory cells including macrophage, the activation and proliferation of HSCs, and the release of fibrogenic mediators [20]. AST and ALT are the aminotransferase in liver cells. They are cytoplasmic in nature, but upon liver injury these enzymes enter into the circulatory system due to altered permeability of membrane. So the elevated serum ALT and AST levels were indicative of hepatotoxicity. In addition, excessive deposition of extracellular matrix components such as collagen protein, proteoglycan and osamine protein is also an important factor in liver fibrosis. HA is an important composition of Connective tissue matrix, which participate to form proteoglycan. HyP is mainly in collagen protein, thus, HA, LN and HyP are an important index to appraisal hepatic fibrosis [21]. In our present study, CCl_4_ caused a significant elevation of serum levels of ALT and AST in rats, and pretreatment with Matrine salvianolic acid B salt significantly attenuated the increased activities of ALT and AST. Moreover, liver index and levels of serum HA, LN, Hyp were increased in CCl_4_-treated rats, and Matrine salvianolic acid B salt markedly decreased the levels of serum HA, LN, Hyp and liver index, suggesting its hepatoprotective effects. Histopathological observations further confirmed that liver injury induced by CCl_4_ was largely prevented by pretreatment of Matrine salvianolic acid B salt,for it has been demonstrated that Matrine salvianolic acid B salt attenuates the necro-inflammatory and fibrogenic effects of CCl_4_, histopathologically.

CCI_4_ damages hepatocellular membrane via lipid peroxidation, and this is followed by the release of inflammatory mediators from the activated inflammatory cells which are thought to potentiate CCl_4_-induced hepatic injury [2,22]. In the present study, the level of lipid peroxide (MDA) in liver tissue was increased and the activities of SOD and GSH were diminished in the CCI_4_-treated rats. However, pretreatment with Matrine salvianolic acid B salt reduced the amount of MDA and increased the activities of SOD and GSH. Together these evidences suggest that the hepatoprotective effects of Matrine salvianolic acid B salt might be in part due to ability to protect biomembrance against the formation of lipid peroxidation.

It is well known that activation of HSCs plays a key role in the pathogenesis of liver fibrosis [3,20]. In our study, CCl_4_ enhanced expression of α-SMA, while the administration of Matrine salvianolic acid B salt prevented the development of fibrosis by inhibiting the activation of HSCs, which has been supported by the reduction in the activation of HSCs as seen by immunohistochemical staining of α-SMA. It has been reported that the activation of HSCs is associated with the elevation of NF-κB activity, and it is well known that cytokines including TGF-β_1_ activate NF-κB signaling [23]. TGF-β_1_ is the most important profibrotic cytokine, and it enhances the type I procollagen synthesis [24,25]. Previous study reported that, proliferation of fibroblast and production of collagen are enhanced by growth factors such as TGF-β_1_. It has been reported that anti-TGF-β_1_ antibody ameliorates concanavalin A-induced hepatic fibrosis [26]. In our study, administration of Matrine salvianolic acid B salt relatively reduced the increased levels of TGF-β_1_ induced by CCl_4_ and therefore, it may be speculated that the anti-fibrotic effect of Matrine salvianolic acid B salt may be mediated by its suppressory effect on TGF-β_1_.

In conclusion, it seems that administration of Matrine salvianolic acid B salt is effective in preventing necro-inflammation and fibrogenesis in hepatic fibrosis induced by CCl_4_. Moreover, Matrine salvianolic acid B salt is a newly prepared compound. This complexes containing one molecular of Salvianolic acid B and one molecular of alkaloids (Matrine). In our study, we can also clearly see the hepatoprotective effect of Matrine salvianolic acid B salt is better than the two monomer componds, indicating Matrine salvianolic acid B salt might be a valuable hepatoprotective agent.

The hepatoprotective effect of Matrine salvianolic acid B salt treatment against CCl_4_-induced hepatic injury seems to be related to the inhibition of lipid peroxidation and levels of TGF-β_1_, the most important inflammatory mediator. However, fibrogenesis may occur as a consequence of an ongoing inflammatory process. Therefore, it is difficult to speculate whether the attenuation of fibrosis by Matrine salvianolic acid B salt is merely a reflection of direct effect on fibrogenesis itself. Further experimental studies are necessary to determine the role and mechanisms of Matrine salvianolic acid B salt in the treatment of hepatic fibrogenesis.

## Authors’ contributions

The author, Hong-Ying Gao carried out the hepatoprotective effect of Matrine salvianolic acid B salt on carbon tetrachloride (CCl_4_)-induced hepatic fibrosis in rats, participated in the analysis of the data and drafted the manuscript. All authors read and approved the final manuscript.

## Competing interests

The authors declare that they have no competing interests.

## Supplementary Material

Additional file 1 Hepatoprotective effect of Matrine salvianolic acid B salt on Carbon Tetrachloride-Induced Hepatic Fibrosis. Click here for file
