# Supplementary material for: Hepatoprotective effect of Matrine salvianolic acid B salt on Carbon Tetrachloride-Induced Hepatic Fibrosis
Source: J Inflamm (Lond). 2012 May 4;9:16. doi: 10.1186/1476-9255-9-16 (PMC3404020; doi:10.1186/1476-9255-9-16)
Supplement: Additional file 1 — Hepatoprotective effect of Matrine salvianolic acid B salt on Carbon Tetrachloride-Induced Hepatic Fibrosis. [file 1476-9255-9-16-S1.doc]

**Innovation**

Matrine is an alkaloid found in kinds of *Sophora* plants mainly including *Sophora flavescens*, *Sophora alopecuroides* and *Sophora subprotrata*. It has a wide range of pharmacological actions, including anti-inflammatory, analgesic, antiarrhythmic, antitumour, antifibrotic, anti-diarrhea and immunosuppressive effects. In recent years, matrine has been used in the treatment of chronic liver disease and has a significant effect on the inhibition of liver fibrosis. Salvianolic acid B is one of the water-soluble components from Traditional Chinese Medical *Salvia miltiorrhiza*. Previous studies have shown that the chemical is effective in improving liver function; alleviating ischemic damage, antioxidation, antihepatotoxity, anticoagulation and antithrombus activity. The newly prepared Matrine salvianolic acid B salt is the synthetic compound by acid-base reaction using Matrine and Salvianolic acid B, which is first reported in this article. Previous studies have shown its protective effect against acute hepatotoxicity by reducing serum AST and ALT levels induced by the treatment of CCl_4_, Thioacetamide(TTA) and D-Galactosamine (D-GalN) respectively in mice. The present study was to investigate the protective effects of Matrine salvianolic acid B salt on the liver fibrosis induced by CCl_4_.

**Illustrate**

Matrine salvianolic acid B salt

Liver tissue Index

SOD

GSH

MDA

Histopathological evaluation

Immunohistological analysis

Serum Index

ALT

AST

HA

LN

HyP

HE

Masson

TGF-β_1_

α-SMA
